# Antibiotic prescribing for acute respiratory infections during the coronavirus disease 2019 (COVID-19) pandemic: Patterns in a nationwide telehealth service provider

**DOI:** 10.1017/ice.2023.292

**Published:** 2024-06

**Authors:** Jeffrey A. Linder, Stephen D. Persell, Marcella A. Kelley, Mark Friedberg, Noah J. Goldstein, Tara K. Knight, Katrina M. Kaiser, Jason N. Doctor, Wendy J. Mack, Jason Tibbels, Bridget McCabe, Steve Haenchen, Daniella Meeker

**Affiliations:** 1 Division of General Internal Medicine, Northwestern University Feinberg School of Medicine, Chicago, Illinois; 2 Center for Primary Care Innovation, Institute for Public Health, Northwestern University Feinberg School of Medicine, Chicago, Illinois; 3 Schaeffer Center for Health Policy & Economics, University of Southern California, Los Angeles, California; 4 School of Pharmacy, University of Southern California, Los Angeles, California; 5 Blue Cross Blue Shield of Massachusetts, Boston, Massachusetts; 6 Anderson School of Management, University of California at Los Angeles, Los Angeles, California; 7 Keck School of Medicine, University of Southern California, Los Angeles, California; 8 Teladoc Health, Inc, Purchase, New York; 9 Yale School of Medicine, Yale University, New Haven, Connecticut

## Abstract

We examined 3,046,538 acute respiratory infection (ARI) encounters with 6,103 national telehealth physicians from January 2019 to October 2021. The antibiotic prescribing rates were 44% for all ARIs; 46% were antibiotic appropriate; 65% were potentially appropriate; 19% resulted from inappropriate diagnoses; and 10% were related to coronavirus disease 2019 (COVID-19) diagnosis.

Unnecessary outpatient antibiotic prescribing for acute respiratory infections (ARIs) contributes to antibiotic resistance, *Clostridioides difficile* infections, and adverse drug events.^1^ Outpatient antibiotic prescribing in the United States decreased marginally prior to the coronavirus disease 2019 (COVID-19) pandemic.^2^ The first year of the pandemic was associated with dramatically fewer visits for non–COVID-19 respiratory conditions and a large decrease in antibiotic use.^3^

At the same time, the uptake of telehealth, including for urgent-care services for ARIs, has surged, and telehealth antibiotic prescribing has come under scrutiny.^4^ Unlike other urgent- and emergent-care in-person settings, physical examinations are limited in what you can see and hear (eg, no palpation available through the internet). Also, laboratory tests are available for order to national labratory vendor collection sites, but point-of-care testing is not available in virtual visits. To describe telehealth antibiotic prescribing and to inform evidence-based outpatient telehealth antibiotic stewardship programs, we examined recent trends in telehealth encounters, antibiotic prescribing, and the appropriateness of antibiotic prescribing for ARIs and COVID-19 telehealth encounters.

## Methods

We conducted an observational study of encounters to a large US telehealth service by patients of all ages between January 2019 and October 2021. Of all episodic urgent-care consultations, we focused on encounters with *International Classification of Diseases, 10th Edition* (ICD-10) codes for acute respiratory infections, including COVID-19 (Supplement 1 Appendix A online). Most patients access the telehealth services as part of employee or insurance benefits. Patient preference and technology dictated whether the visit was via phone or video. Encounters were assigned to the first available clinician or by appointment to clinicians responsible for evaluation, diagnosis, and management of patient symptoms. Clinicians followed standard procedures for intake assessments, medical history taking, visual or audio observations of physical conditions, emergency escalation protocols, and progress note documentation. All clinician, diagnosis, prescription, and referral data were captured in the telehealth service electronic medical record.

We considered ARI visits with a diagnosis of sinusitis, bronchitis, influenza, otitis media, nasopharyngitis, upper respiratory infection, or COVID-19. Encounters with a COVID-19 diagnosis code were categorized as COVID-19 encounters; ARI encounters without a COVID-19 code were considered non–COVID-19 encounters.

Using an established scheme based on all encounter diagnoses, we defined each encounter as antibiotic always appropriate, potentially appropriate, or inappropriate.^5^ COVID-19 diagnoses were assigned beginning February 2020.

We calculated annual means of diagnosis types, antibiotic prescribing, and antibiotic appropriateness and plotted weekly rates over the same period. All analyses were performed using the R tidyverse suite (R Studio, R Foundation for Statistical Computing, Vienna, Austria). Given the large sample size, we did not perform formal statistical testing and considered differences of 5% or greater as clinically significant.

## Results

In 2019, ARI encounters accounted for 43% of all episodic urgent care consults, decreasing to 23% in 2021 (Supplement 2 Fig. A1 online). There were 3,046,538 ARI encounters with 6,103 telehealth physicians from January 2019 to October 2021 (Table 1). From 2020 to 2021, the proportion of COVID-19 encounters increased from 7.4% to 11.4%. Among ARI visits, 20% used video and most patients’ complaints were resolved without a referral to another site of care (Supplement 3, Table A1 online). Almost all encounters (92%) were with the first available clinician.

**Table 1.** Counts of Consultations and Antibiotic Prescribing by Appropriateness Categories

	Overall			Jan–Dec 2019			Jan–Dec 2020			Jan–Oct 2021		
Antibiotic Appropriateness Category	All ARI	Non–COVID-19	COVID-19	All ARI	Non–COVID-19	COVID-19	All ARI	Non–COVID-19	COVID-19	All ARI	Non-COVID-19	COVID-19
**Antibiotic Prescribing Rate, %**												
All categories combined	44	48	10	46	46	…	41	47	8	47	52	12
Always appropriate	46	48	15	47	47	…	43	46	11	50	52	20
Potentially appropriate	65	66	21	47	47	…	61	64	18	68	71	24
Inappropriate	19	22	7	20	20	…	18	22	6	19	22	9
**Consultations, N (%)**												
All	3,046,538	2,762,620	283,918	974,454	974,454	…	1,200,406	1,030,734	169,672	871,678	757,432	114,246
Always	94,160 (3)	89,231 (3)	4,929 (2)	24,275 (2)	24,275 (2)	…	36,439 (3)	33,811 (3)	2,628 (2)	33,446 (4)	31,145 (4)	2,301 (2)
Potentially	1,639,194 (54)	1,586,276 (57)	52,918 (19)	548,845 (56)	548,845 (56)	…	624,287 (52)	593,542 (58)	30,745 (18)	466,062 (53)	443,889 (59)	22,173 (19)
Inappropriate	1,313,184 (43)	1,087,113 (39)	226,071 (80)	401,334 (41)	401,334 (41)	…	539,680 (45)	403,381 (39)	136,299 (80)	372,170 (43)	282,398 (37)	89,772 (79)

Note. ARI, acute respiratory infection.

**Figure 1.**
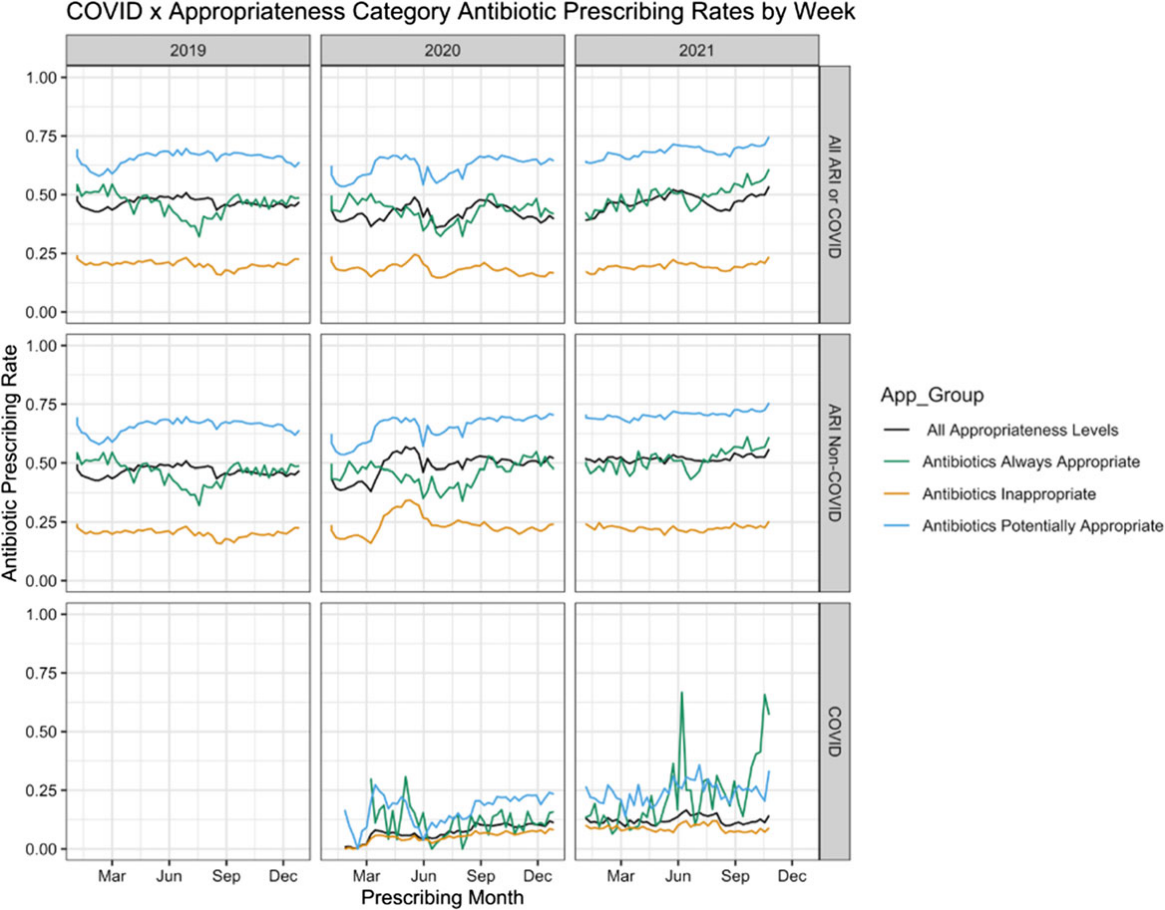
Antibiotic prescribing over time for acute respiratory infection (ARI) encounters, January 2019–October 2021. Weekly antibiotic prescribing rates for encounters with any ARI or COVID-19 diagnosis (first row), ARIs that do not include COVID-19 (second row), and COVID-19 encounters where COVID-19 diagnosis was present (third row). Antibiotics are considered appropriate or potentially appropriate in COVID-19 encounters where an appropriate or potentially appropriate diagnosis was present in the same encounter.

The antibiotic prescribing rates were 44% for all ARIs; 46% were antibiotic appropriate; 65% were potentially appropriate; 19% resulted from inappropriate diagnoses; and 10% were related to coronavirus disease 2019 (COVID-19) diagnosis (Table 1). Antibiotic prescribing rates decreased at the start of the pandemic and rebounded quickly (Fig. 1). Appropriate antibiotic prescribing was similar between 2019 and 2020, with a decrease in the summer months, though this decrease was less pronounced in 2021. Antibiotic prescribing for COVID-19 encounters without concurrent ARI diagnoses where antibiotics are appropriate was rare at the start of the pandemic but increased over time, from 5.8% at the start of the pandemic to 8.9% at the end of the study period (Fig. 1). Notably, antibiotics were prescribed for COVID-19 less than half as frequently as other ARIs for which antibiotics are never indicated, and prescriptions for “potentially appropriate” categories like pharyngitis were higher than other appropriateness groups.

## Discussion

The proportion of ARI visits in telehealth shifted during the pandemic, disrupting seasonal trends in diagnoses. However, overall, antibiotic prescribing rates remained proportionally consistent within diagnostic categories over time. The exception to this occurred in the first 2 quarters of 2020, when a steep decline and subsequent rebound in inappropriate prescribing was observed. This pattern may be attributed to more conservative practices early in the pandemic, practitioner training, or changes in workforce composition. The proportion of ARI encounters steadily increased through 2020 and 2021 but did not reach seasonal levels comparable to those of 2019, even when including COVID-19. This difference may be attributed to either changes in scope of practice for telehealth or changes in etiology. Consistent with findings in other settings, antibiotic prescribing for potentially appropriate diagnoses was higher than either appropriate or inappropriate diagnoses.^1^ Overall telehealth-based antibiotic prescribing rates increased slightly between 2019 and 2021, partially explained by increases in diagnoses for which antibiotics were appropriate. This change may have been due to changes in the mixture of patients using telehealth, changes in etiology, or changes in coding practices. Future work may further explore these details, as well as differences across subgroups, such as age, preferred visit modality, or patient and physician characteristics.

For several conditions, studies have shown comparable quality between telehealth and in-person care.^6,7^ Telehealth, and specifically urgent-care telehealth, is a growing setting for treatment of ARIs; antibiotic stewardship programs that are tailored to telehealth may be warranted. Patient expectations and satisfaction have been postulated as drivers of demand (or physicians’ perceived demand) for antibiotics.^8,9^ These efforts may require focus on communication skills or performance measurement programs that balance satisfaction with quality incentives for ARIs. The fact that telehealth physicians rarely prescribed antibiotics for COVID-19 without changing inappropriate prescribing rates for other infections may shed some light on context-dependent factors. Results should be interpreted in the context of issues associated with availability of diagnostic testing in telehealth and coding practices associated with all EHR data.

Between 2019 and 2021, temporal and seasonal patterns for acute respiratory infections and COVID-19 in telehealth varied, but annual proportions of antibiotic prescribing appropriateness were stable. However, in the first quarter of the pandemic, we observed reduced inappropriate antibiotic prescribing that rebounded to seasonally high levels by the summer of 2020. These types of variations indicate that there are opportunities to improve antibiotic stewardship in telehealth, particularly for ARIs.

## Supporting information

Linder et al. supplementary material 1 Linder et al. supplementary material

Linder et al. supplementary material 2 Linder et al. supplementary material

## Supplementary material

To view supplementary material for this article, please visit https://doi.org/10.1017/ice.2023.292
