# Supplementary figures and images for: Antibiotic prescribing for acute respiratory infections during the coronavirus disease 2019 (COVID-19) pandemic: Patterns in a nationwide telehealth service provider
Source: Infect Control Hosp Epidemiol. 2024 Feb 8;45(6):777–80. doi: 10.1017/ice.2023.292 (PMC11102822; doi:10.1017/ice.2023.292)

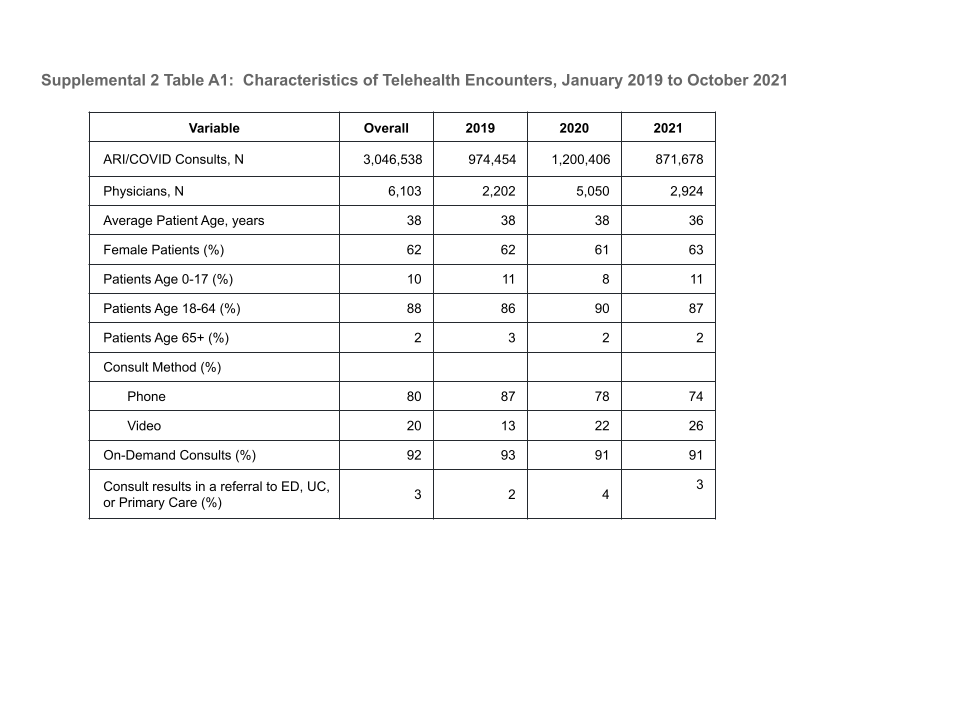

Supplement: Linder et al. supplementary material 2 — Linder et al. supplementary material [file S0899823X23002921sup002.tif]
